# Influenza A (H1N1) 2009: Impact on Frankfurt in due consideration of health care and public health

**DOI:** 10.1186/1745-6673-5-10

**Published:** 2010-04-26

**Authors:** Sabine Wicker, Holger F Rabenau, Harald Bias, David A Groneberg, René Gottschalk

**Affiliations:** 1 Occupational Health Service, Hospital of the Johann Wolfgang Goethe-University, Theodor-Stern-Kai 7, 60590 Frankfurt am Main, Germany; 2 Institute of Medical Virology, Hospital of the Johann Wolfgang Goethe-University, Paul-Ehrlich-Str. 40, 60596 Frankfurt am Main, Germany; 3 Institute of Occupational Medicine, Charité - Universitätsmedizin Berlin, Free University and Humboldt-University Berlin, Thielalllee 69-73, 14195 Berlin, Germany; 4 Health Protection Authority, City of Frankfurt am Main, Breite Gasse 28, 60313 Frankfurt am Main, Germany

## Abstract

**Background:**

In April 2009 a novel influenza A H1N1/2009 virus was identified in Mexico and in the United States which quickly spread around the world. Most of the countries established infection surveillance systems in order to track the number of (laboratory-confirmed) H1N1 cases, hospitalizations and deaths.

**Methods:**

The impact of the emergence of the novel pandemic (H1N1) 2009 virus on Frankfurt was statistically evaluated by the Health Protection Authority, City of Frankfurt am Main.

Vaccination rates of the health care workers (HCWs) of the University Hospital Frankfurt were measured by the Occupational Health Service.

**Results:**

Although the virulence of pandemic (H1N1) 2009 seems to be comparable with seasonal influenza, a major patient load and wave of hospital admissions occurred in the summer of 2009.

Even though the 2009 vaccination rate of the University Hospital Frankfurt (seasonal influenza [40.5%], swine flu [36.3%]) is better than the average annual uptake of influenza vaccine in the German health care system (approximately 22% for seasonal and 15% for swine flu), vaccination levels remain insufficient.

However, physicians were significantly (p < 0.001) more likely to have been vaccinated against swine flu and seasonal influenza than nurses.

**Conclusions:**

The outbreak of the pandemic (H1N1) 2009 in April 2009 provided a major challenge to health services around the world. Nosocomial transmission of H1N1/2009 has been documented. Present experience should be used to improve pandemic preparedness plans and vaccination programs ought to target as many HCWs as possible.

## Background

When the pandemic (H1N1) 2009 flu outbreak began in April 2009, the Centers for Disease Control (CDC) in U.S.A. and the Robert Koch Institute (RKI) in Germany began tracking and reporting the number of laboratory-confirmed influenza A H1N1/2009 cases, hospitalizations and deaths. These initial case counts (which were discontinued on July 24, 2009 in U.S.A. and on November 14, 2009 in Germany) are thought to represent a significant undercount of the actual number of influenza A H1N1/2009 flu cases, especially in the U.S. [1].

Almost a year later (April, 2010), influenza activity continues to decline or remain low in most countries. Based on FluNet data http://gamapserver.who.int/GlobalAtlas/home.asp collected by 32 countries from February 6 - 13, 2010, 48.3% of specimens testing positive for influenza were typed as influenza A and 51.7% as influenza B. In nearly all countries where influenza infection has been reported, the influenza A H1N1/2009 continues to predominate among all subtyped influenza A viruses. Out of all subtyped influenza A viruses, 90% were influenza A H1N1/2009 positive [2].

Through April 13, 2010, a total of 226,125 infections with pandemic (H1N1) 2009 had been reported in Germany, including 253 deaths [3].

The purpose of the present study was to ascertain the impact of the emergence of the novel influenza A H1N1/2009 virus on Frankfurt, a metropolis with the largest airport in Germany. Furthermore, we assessed vaccination rates of health care workers (HCWs) of the University Hospital Frankfurt.

## Methods

Frankfurt am Main has 675,729 inhabitants making it the fifth largest city of Germany. Frankfurt Airport plays a key role in international air transportation. With more than 50.9 million passengers in 2009, it ranks eighth in the league table of the world's largest airports. In Europe, it is number three in terms of passengers after London-Heathrow and Paris-Charles de Gaulle. Nowadays infectious diseases and pandemics are primarily spread through aviation, for this reason there is a high risk of introducing emerging infectious diseases in the Rhein-Main region [4].

In Frankfurt am Main there are approximately 604,500 workplaces, therefore Frankfurt holds the highest job density per inhabitant in Germany. Approximately 89.2% are employed in the service sector and about 10.7% in production industries [5].

The duties and responsibilities of the public health service in Frankfurt are assumed by the Health Protection Authority of the City of Frankfurt am Main. The local health authority advises the population on the prevention of infectious diseases and on the prophylaxis of transmission of infections. People with suspected or confirmed influenza A H1N1/2009 have been reported to the Municipal Health Protection Authorities since April 30, 2009. The Office monitors the number of patients who have been detected as confirmed, probable or suspicious cases, patients who require hospitalization and the fatal causalities as well.

The Health Protection Authority of the City of Frankfurt am Main co-ordinated, on behalf of the Ministry of Health of Hesse, the swine-flu vaccination campaign in Frankfurt. Vaccinations have been administered in the office since the end of October 2009 in the following order: prioritized risk groups (HCWs, fire-fighters etc.), patients with chronic diseases, pregnant women, household contact of non-vaccinated risk groups, healthy children and young adults up to 24, healthy adults from the age range 25-59 and lastly people over the age of 60.

There are 16 hospitals in Frankfurt; the biggest one is the Frankfurt University Hospital, which is a 1,169-bed hospital with 3,900 employees (including 726 physicians, 1,300 nurses and nursing assistants) working in 24 medical departments and research facilities.

From October 2009 to March, 2010, the Occupational Health Service of the University Hospital offered seasonal influenza and swine flu vaccinations free of charge to HCWs.

The Occupational Health Service, the Institute of Medical Microbiology and Infection Control and the Institute of Medical Virology provided recommendations for infection control of influenza A H1N1/2009 in the University Hospital in co-operation with the Health Protection Authority.

### Statistical analysis

For statistical analysis, data was inserted into a Microsoft Excel database file. This file represented the basis for the detailed analysis using standard MS Excel capabilities. *P *values (χ^2 ^test - two-tailed χ^2^-test, Yates rectified) were calculated using the BiAS program for Windows 8.3 (Epsilon Verlag, Hochheim Darmstadt 2007). *P *values < 0.05 were defined as statistically significant.

## Results

**Figure 1:**
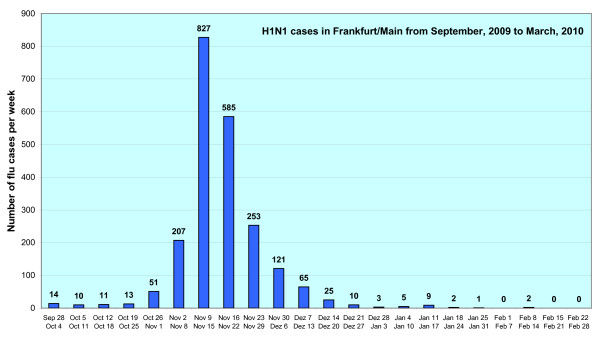
**Reported cases of influenza A H1N1/2009 (n = 2,214) in Frankfurt am Main**. Starting from November 16, 2009 only laboratory confirmed cases were counted.

By March 5, 2010, in total 2,214 cases of influenza A/H1N1/2009 had been confirmed and had been reported to the Health Protection Authority of the City of Frankfurt am Main (see Figure 1). These cases resulted in 4 known deaths.

**Figure 2:**
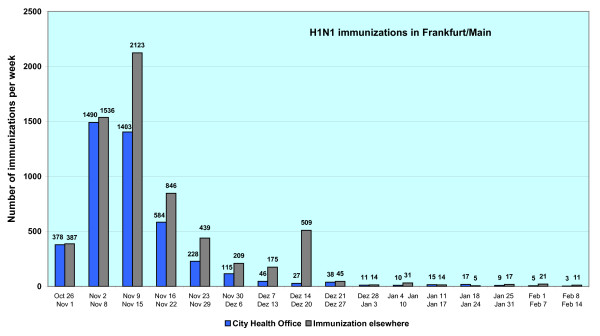
**Number of H1N1/2009 immunizations in Frankfurt am Main (n = 10,761)**. Overall, 4,379 vaccinations were administered by the Health Protection Authority of Frankfurt am Main. In total 6,382 vaccinations were administered by other immunization stations (e.g. University Hospital Frankfurt).

Overall 10,761 H1N1 vaccinations were administered in Frankfurt am Main (see Figure 2). This led to a rather low vaccination rate (< 1.6%) of the inhabitants of Frankfurt (n = 675,729). Unfortunately, the vaccination rate of the inhabitants of Frankfurt cannot be calculated exactly, owing to the fact that a proportion of immunizations had been given to persons whose main residence is not Frankfurt am Main. These vaccinees were vaccinated e.g. at their workplaces.

From October 2009 to the end of February 2010, overall, 40.5% (n = 1,579/3,900) of the HCWs of the University Hospital Frankfurt were vaccinated against seasonal influenza, and 36.3% (n = 1,416/3,900) were vaccinated against influenza A/H1N1/2009 ("swine flu").

The average age of an employee of the University Hospital is 42 years, the average age of the seasonal influenza vaccinees was 39.6 (range: 19-67 years), the average age of the swine flu vaccinees was 38.2 (range: 20-64 years).

Physicians (n = 586/726) were significantly more likely to have been vaccinated against swine flu than nurses (n = 393/1300) (80.7% versus 30.2%, respectively; p < 0.001). Roughly the same was shown for seasonal influenza.

Men and women were analyzed separately, providing an insight into gender-specific determinants of immunization behavior. Table 1 and 2 present vaccination rates according job description and gender, separated for each vaccine and HCW who received both vaccines. In total, 48% of the H1N1 ("swine flu") vaccinees were also given the seasonal flu vaccine and 43% of the seasonal influenza vaccinees received the H1N1 vaccine as well (see Table 2).

**Table 1:** Demographic characteristics of vaccines (separated for each kind of flu vaccine)

	H1N1 ("swine flu")			Seasonal influenza			
	**n**	**[%]**	**p-value**	**n**	**[%]**	**p-value**	

Vaccination rate	1,416/3,900	36.3		1,579/3,900	40.5	→	P < 0.001

Male (n = 1,158)	634	54.7	p < 0.001	574	49.6	p < 0.001	P = 0.013
Female (n = 2,742)	782	28.5	p < 0.001	1,005	36.7	p < 0.001	P < 0.001
**Job description**							

Physicians (n = 726)	586	80.7	p < 0.001	497	68.5	p < 0.001	P < 0.001
Nurses (n = 1,300)	393	30.2	p < 0.001	432	33.2	p < 0.001	P = 0.100
Medical technicians(n = 850)	101	11.8		171	20.1		
Scientists (n = 224)	176	78.6		127	56.7		
Administrative personnel (n = 300)	93	31.0		154	51.3		
Others (e.g.: Maintenance, catering, workshop, transport) (n = 500)	67	13.4		198	39.6		

**Table 2:** Characteristics of HCWs who received both influenza vaccines (against H1N1/2009 and seasonal flu)

	Proportion of H1N1 ("swine flu") vaccinees who also received seasonal influenza vaccination [%]		
**Vaccination H1N1**	**[n]**	**[%]**	**p-value [%]**

Total (n = 1,416)	679	48.0	

Male (n = 634)	312	49.2	0.393
Female (n = 782)	367	46.9	0.393
**Job description**			

Physicians (n = 586)	331	56.5	0.008
Nurses (n = 393)	188	47.8	0.008
Medical technicians (n = 101)	45	44.6	
Scientists (n = 176)	52	29.5	
Administrative personnel (n = 93)	39	41.9	
Others (e.g.: Maintenance, catering, workshop, transport) (n = 67)	24	35.8	

Overall, 49.2% (312/634) of the male H1N1 vaccinees also received a seasonal flu vaccination and 54.4% (312/574) of the seasonal flu vaccines received a H1N1/2009 vaccination (p = 0.074).

While there is no significant difference in the proportion of male and female H1N1/2009 vaccinated HCWs who also received a seasonal flu vaccination (p = 0.393), a gender-specific difference could have been demonstrated for seasonal flu vaccinees who also received a H1N1/2009 vaccination (male 54.4% [312/574] versus female 36.5% [367/1,005]; p < 0.001).

## Discussion

Evidence from the past few months demonstrates that the influenza A H1N1/2009 virus has rapidly established itself and is now the dominant influenza strain in most parts of the world [2,6].

Influenza viruses are highly contagious; the basis reproduction number (R_0_) of influenza A H1N1/2009 was estimated to be between 1.4 and 1.6 [7]. If the R_0 _is greater than 1, a pandemic might occur [6].

In the City of Frankfurt am Main 2,214 probable and confirmed cases were notified to the Health Protection Authority. However, this number reflects only a small fraction of the people with the pandemic (H1N1) 2009 influenza infection. Estimated numbers of unknown cases will be distinctly higher for a variety of reasons, especially since not all patients seek medical care due to a mild course of the disease. In a study performed between April and July 2009 in the U.S., the estimated numbers of unknown cases were calculated to 79:1 with a 90% probability range of 47-148, which means that one registered flu case correspond with 79 unknown cases [8]. Estimating the numbers of unknown cases to be 70 for the City of Frankfurt am Main a total of nearly 155,000 cases (or 23% of the inhabitants) seems to be realistic assessment.

Fortunately, the majority of cases are considered to be lenient. Fatal causes occur mostly but not exclusively in patients with underlying medical conditions (chronic diseases such as asthma, diabetes, immunosuppression, obesity).

The working environment may be crucial to pandemic preparedness planning [9]. Workplaces are potential sources of disease transmission, and illness and absenteeism might lead to substantial productivity losses and could disrupt the functionality of the health care system [10]. HCWs are at risk of occupational exposure to influenza and may transmit the infection to their patients and co-workers [11,12]. The influenza attack rate among unprotected HCWs might be approximately 60% higher than that of the general population, which would result in substantial absenteeism and morbidity [13]. On account of this, the health care system needs to be aware of the safety of their HCWs because they are at significant risk of becoming infected [14].

Recent data suggest that the influenza A H1N1/2009 virus is transmitted via large particle droplets [15]. Because large droplets remain suspended in the air only for a short time, close contact is a precondition for virus transmission [6]. Occupationally acquired infections of influenza A H1N1/2009 in HCWs have been documented [16]. Unfortunately, nurses (who usually have both closer and longer contact with patients than any other professional group of HCWs) demonstrate flu vaccination rates which are 2 up to 2.5 times lower than the vaccination rates of physicians (see Table 1).

Vaccination seems to be the best defense against high infection rates among susceptible and vulnerable people. Nevertheless, compliance rates with influenza vaccination among HCWs and the general population remain low [17-19].

Current data shows that by the end of December 2009 as few as 22% of U.S. HCWs had received the swine flu vaccine [6]. Vaccination rates in Germany are just as low, an estimated 15% of German HCWs have received the swine flu vaccine [17]. Albeit, the vaccination rate of the University Hospital Frankfurt is better, both for swine flu (36.3%), and seasonal influenza (40.5%), than the average annual uptake of the influenza vaccine in the German health care system (approximately 22%). Nevertheless, vaccination levels among HCWs remain insufficient. It is crucial that an effective response to a pandemic as well as a mitigation of the associated morbidity and mortality ought to be predicated on a vaccinated, working, and informed health care population [6].

Compared to other HCWs, nurses have lower flu vaccination rates and seem to be most doubtful of influenza vaccine efficacy and necessity and most afraid of their adverse effects. A study of a large tertiary medical center revealed that nurses had fears and misconception about influenza vaccination despite perceived receipt of adequate information to support good decision-making. Furthermore, nurses judged influenza vaccination as a personal health choice, not as an evidence-based nursing intervention [20].

The pulmonary pathologic findings in fatal causalities caused by influenza A H1N1/2009 virus are similar to findings identified in the 1918 and 1957 pandemics [21]. There is still considerable uncertainty about how the influenza A H1N1/2009 virus will behave over the coming months and years. To achieve data for prediction of the future development an early and consequent surveillance and monitoring system with a standardized and coordinated international information sharing is crucial for the management not only for pandemic influenza but all pandemics [22]. The setting of standards for coping with this should be subject to a municipal or local decision but also established at national and global levels. National authorities need to know how the pandemic is evolving, not only in their own country, but also in neighboring countries and continents [23].

Thus far, in contrast to seasonal influenza viruses, the influenza A H1N1/2009 virus has disproportionately affected young people, and this is where most complications have occurred, particularly in those with pre-existing chronic conditions. At this point of time the virulence of influenza A H1N1/2009 virus is similar to that of seasonal influenza viruses [6]. However, we do not yet know if there might be a change.

### Limitations

To appreciate the results of our study, some potential limitations need to be addressed:

First, the results from a single academic institution or city may not be applicable to other institutions and other geographic regions. Second, the number of HCWs who received either their seasonal flu vaccination or the swine flu vaccination from their general practitioner or other health authorities could not be calculated. Third, the immunization rate and the overall number of cases of the inhabitants of Frankfurt could only be estimated.

## Conclusions

This influenza A/H1N1 pandemic differs in significant aspects from the experiences gained from earlier pandemics. All simulations which predicted the potential course of this pandemic have been wrong. Measures of infection control of the public health authorities are in place and have been proven to be effective irrespective of the specific agents. A practice-based and future-oriented perfect preparation for taking on the challenges of pandemics is considered to be indispensable. Due to informational needs of the public and employees, professional, constant and reliable risk communication is crucial to successfully cope with pandemics.

## Conflict of interests

The views in this article are the personal views of the authors and do not necessarily represent the views of the professional organizations or institutions within which we are members.

The authors declare that they have no competing interests.

## Authors' contributions

SW and RG drafted the manuscript.

SW, HFR and RG conceived the study and the study design, performed the analysis and interpretation of the data.

DAG and HB: scientific supervision, revised the manuscript critically for important intellectual content.

All authors read and approved the final manuscript.

## References

[B1] Centers for Disease Control and Prevention (CDC) CDC Estimates of 2009 H1N1 Influenza Cases, Hospitalizations and Deaths in the United States, April 2009 - January 16, 2010 http://www.cdc.gov/h1n1flu/estimates_2009_h1n1.htm

[B2] Centers for Disease Control and Prevention (CDC) 2009 H1N1 Flu: International Situation Update February 26, 2010 http://www.cdc.gov/h1n1flu/updates/international/

[B3] Robert Koch Institut Arbeitsgemeinschaft Influenza http://www.rki.de/cln_179/nn_205760/DE/Content/InfAZ/I/Influenza/IPV/IPV__Node.html?__nnn = true

[B4] Gaber W Goetsch U Diel R Doerr HW Gottschalk R Screening for Infectious Diseases at International Airports: The Frankfurt Model Aviat Space Environ Med 2009 80 595 600 10.3357/ASEM.2360.2009 19601499

[B5] Statistisches Jahrbuch: Statistical portrait Frankfurt am Main 2008 http://www.frankfurt.de/sixcms/media.php/678/Statistisches_Portrait_2009x.pdf

[B6] Sullivan SJ Jacobson RM Dowdle WR Poland GA 2009 H1N1 Influenza Mayo Clin Proc 2010 85 64 76 10.4065/mcp.2009.0588 20007905 PMC2800287

[B7] Fraser C Donnelly CA Cauchemez S Hanage WP Van Kerkhove MD Hollingsworth TD Griffin J Baggaley RF Jenkins HE Lyons EJ Jombart T Hinsley WR Grassly NC Balloux F Ghani AC Ferguson NM Rambaut A Pybus OG Lopez-Gatell H Alpuche-Aranda CM Chapela IB Zavala EP Guevara DM Checchi F Garcia E Hugonnet S Roth C WHO Rapid Pandemic Assessment Collaboration Pandemic potential of a strain of influenza A (H1N1): early findings Science 2009 324 1557 1561 10.1126/science.1176062 19433588 PMC3735127

[B8] Reed C Angulo FJ Swerdlow DL Lipsitch M Meltzer MI Jernigan D Finelli L Estimates of the Prevalence of Pandemic (H1N1) United States, April-July 2009 Emerg Infect Dis 2009 15 2004 2007 10.3201/eid1512.091413 19961687 PMC3375879

[B9] Blake KD Blendon RJ Viswanath K Employment and compliance with pandemic influenza mitigation recommendations Emerg Infect Dis 2010 16 212 218 20113549 10.3201/eid1602.090638 PMC2958001

[B10] Lee BY Brown ST Cooley PC Zimmerman RK Wheaton WD Zimmer SM Grefenstette JJ Assi TM Furphy TJ Wagener DK Burke DS A computer simulation of employee vaccination to mitigate an influenza epidemic Am J Prev Med 2010 38 247 257 10.1016/j.amepre.2009.11.009 20042311 PMC2833347

[B11] Blachere FM Lindsley WG Pearce TA Anderson SE Fisher M Khakoo R Meade BJ Lander O Davis S Thewlis RE Celik I Chen BT Beezhold DH Measurement of airborne influenza virus in a hospital emergency department Clin Infect Dis 2009 48 438 440 10.1086/596478 19133798

[B12] Mermel LA Preventing the spread of influenza A H1H1 2009 to health-care workers Lancet Infect Dis 2009 9 723 724 10.1016/S1473-3099(09)70299-3 19892599

[B13] Cooley P Lee BY Brown S Cajka J Chasteen B Ganapathi L Stark JH Wheaton WD Wagener DK Burke DS Protecting health care workers: a pandemic simulation based on Allegheny County Influenza other respi viruses 2010 4 61 72 10.1111/j.1750-2659.2009.00122.x 20167046 PMC2894576

[B14] Shine KI Rogers B Goldfrank LR Novel H1N1 influenza and respiratory protection for health care workers N Engl J Med 2009 361 1823 1825 10.1056/NEJMp0908437 19797278

[B15] Maines TR Jayaraman A Belser JA Wadford DA Pappas C Zeng H Gustin KM Pearce MB Viswanathan K Shriver ZH Raman R Cox NJ Sasisekharan R Katz JM Tumpey TM Transmission and pathogenesis of swine-origin 2009 A(H1N1) influenza viruses in ferret and mice Science 2009 325 484 487 19574347 10.1126/science.1177238 PMC2953552

[B16] Wicker S Rabenau HF Bickel M Wolf T Brodt R Brandt C Berger A Doerr HW Lehmann R Novel Influenza H1N1/2009: Virus transmission among health care worker Dtsch Med Wochenschr 2009 134 2443 2446 German 10.1055/s-0029-1242012 19908176

[B17] Rieser S Swine flu: Criticism on vaccination-reluctance of physicians Dtsch Arztebl 2010 107 1 2 German

[B18] Singleton JA Santibanez TA Lu PJ Ding H Euler GL Armstrong GL Bell BP Town M Balluz L Interim results: Influenza A (H1N1) 2009 Monovalent vaccination coverage - United States, October - December 2009 MMWR 2010 59 1 5 20094027

[B19] Poland GA Tosh P Jacobson RM Requiring influenza vaccination for health care workers: seven truths we must accept Vaccine 2005 23 2251 2255 10.1016/j.vaccine.2005.01.043 15755605

[B20] Rhudy LM Tucker SJ Ofstead CL Poland GA Personal choice or evidence-based nursing intervention: nurses' decision-making about influenza vaccination Worldviews Evid Based Nurs 2010 in press 20367806 10.1111/j.1741-6787.2010.00190.x

[B21] Gill JR Sheng ZM Ely SF Guinee DG Beasley MB Suh J Deshpande C Mollura DJ Morens DM Bray M Travis WD Taubenberger JK Pulmonary pathologic findings of fatal 2009 pandemic influenza A/H1N1 viral infections Arch Pathol Lab Med 2010 134 235 243 20121613 10.5858/134.2.235 PMC2819217

[B22] World Health Organization (WHO) Human infection with pandemic (H1N1) 2009 virus: updated interim WHO guidance on global surveillance (2009) http://www.who.int/csr/disease/swineflu/guidance/surveillance/WHO_case_definition_swine_flu_2009_04_29.pdf

[B23] World Health Organization (WHO) Reducing transmission of pandemic (H1N1) 2009 in school settings (2009) http://www.who.int/csr/resources/publications/reducing_transmission_h1n1_2009.pdf

